# Neurocysticercosis in a Non-endemic Setting: A Report of Three Cases

**DOI:** 10.7759/cureus.114595

**Published:** 2026-08-16

**Authors:** Meryem El Marbouh, Houyam Tibar, Farouk Handaoui, Aziza El Malti, Hajar Naciri Darai, Dahab Ouhabi, Ali Benomar, Wafa Regragui

**Affiliations:** 1 Department of Neurology B and Neurogenetics, Specialties Hospital, Ibn Sina Teaching Hospital, Faculty of Medicine and Pharmacy of Rabat, Mohammed V University in Rabat, Rabat, MAR; 2 Department of Neurology and Interventional Neuroradiology, Cheikh Zaid International University Hospital, Abulcasis International University of Health Sciences, Rabat, MAR

**Keywords:** morocco, neurocysticercosis, neuroimaging, seizures, taenia solium

## Abstract

Neurocysticercosis can present a diagnostic challenge in settings where it is not routinely suspected. Although endemic in many developing regions, it remains uncommon in Morocco, which may delay diagnosis. We report three cases illustrating its clinical and radiological variability in a non-endemic setting. All patients came from rural areas and presented with seizures, while one also reported chronic headaches and severe fatigue. Neuroimaging revealed predominantly parenchymal lesions at different evolutionary phases, including scolex-containing lesions, calcifications, and a ring-enhancing lesion suggestive of the colloidal phase. Serology may provide supportive evidence, but a negative result does not exclude intraparenchymal neurocysticercosis. These cases emphasize the importance of considering neurocysticercosis in patients with suggestive neurological and radiological findings, even in non-endemic settings.

## Introduction

Neurocysticercosis (NCC) is the neurological form of cysticercosis caused by the larval stage of *Taenia solium* (TS) [1]. Humans develop NCC after ingesting TS eggs through fecal-oral transmission or, less commonly, by autoinfection [1,2]. In contrast, intestinal taeniasis is acquired by consuming undercooked pork containing viable cysticerci, which mature into adult tapeworms in the intestine. These tapeworms shed eggs in the carrier's feces, enabling transmission to the carrier or other individuals and potentially causing cysticercosis, including NCC [3].

Although NCC remains endemic in many regions, particularly in parts of Latin America, sub-Saharan Africa, and Asia [2], it is increasingly recognized in non-endemic settings because of migration, travel, and population mobility [2,4]. In Morocco, a Muslim-majority country where religious and cultural practices generally limit pork consumption, thereby reducing opportunities for TS transmission, NCC is considered uncommon but should not be overlooked. A careful exposure history, including residence or travel in endemic areas, rural living conditions, poor sanitation, and possible contact with a tapeworm carrier, may provide important diagnostic clues.

Diagnosis can be challenging in such settings because the perceived rarity of NCC may reduce clinical suspicion. Clinical manifestations are variable and nonspecific, with seizures being the most common presenting symptom [1]. Neuroimaging is central to the diagnostic workup, as characteristic patterns may indicate lesion viability and evolution. Computed tomography (CT) is particularly useful for detecting calcifications, whereas magnetic resonance imaging (MRI) better identifies viable or degenerating cysts, perilesional edema, and enhancement [1,3].

We report three cases of NCC diagnosed in a non-endemic setting, illustrating the diverse clinical and radiological spectrum of the disease and emphasizing the importance of considering NCC in the differential diagnosis of patients with compatible neurological presentations and relevant epidemiological exposure.

## Case presentation

Case 1

A 53-year-old male farmer with reported contact with wild boars and close contact with individuals who consumed wild-boar meat presented with persistent headaches. Six months earlier, he had been hospitalized in the intensive care unit for febrile meningoencephalitis with altered consciousness and generalized tonic-clonic seizures. He improved after nonspecific antibiotic treatment and was discharged on sodium valproate 500 mg twice daily.

Some days later, he developed progressive diffuse headaches resistant to symptomatic treatment, associated with weight loss and severe fatigue, with a Fatigue Severity Scale (FSS) score of 6/7. He reported no further seizures. On admission, he was conscious and hemodynamically stable, with a blood pressure of 126/67 mmHg, a heart rate of 72 beats/min, and a body temperature of 36.4°C. Neurological and general examinations, as well as ophthalmological examination with fundoscopy, were otherwise unremarkable.

**Figure 1:**
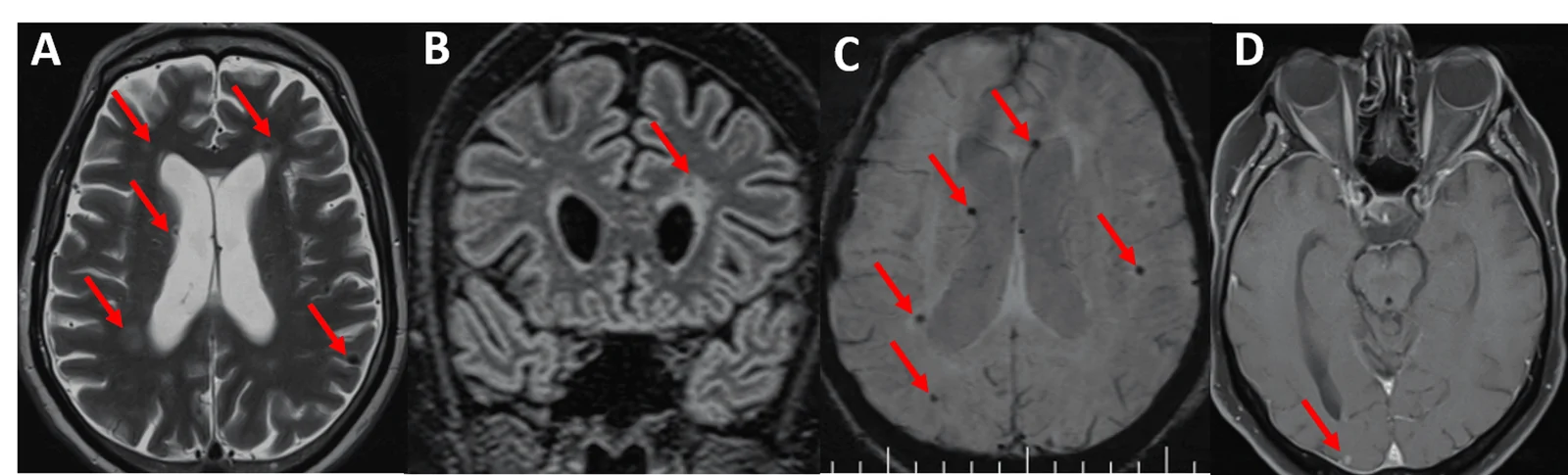
Brain magnetic resonance imaging (A) T2-weighted imaging shows rounded hypointense cortico-subcortical lesions surrounded by edema. (B) Coronal fluid-attenuated inversion recovery imaging demonstrates a periventricular lesion with a dot sign corresponding to the parasite scolex. (C) Multiple cortico-subcortical lesions demonstrate signal void on susceptibility-weighted imaging. (D) After contrast administration, enhancement of an occipital parasagittal lesion is seen.

Brain MRI showed multiple rounded cortico-subcortical and thalamic lesions, hypointense on T2-weighted, fluid-attenuated inversion recovery (FLAIR), and diffusion-weighted imaging (DWI) sequences, with signal void on susceptibility-weighted imaging (SWI). One right occipital parasagittal lesion enhanced after contrast administration. Some lesions showed the characteristic "dot sign," corresponding to an eccentric scolex, considered pathognomonic for NCC (Figure 1). Cerebrospinal fluid (CSF) analysis was normal, with negative tuberculosis polymerase chain reaction (PCR) and multiplex PCR. Blood work showed elevated C-reactive protein (CRP) at 126 mg/L. Serum enzyme-linked immunosorbent assay (ELISA) for TS was negative. Screening for ophthalmological localization and subcutaneous calcifications was negative.

Because we identified viable parenchymal lesions on MRI, we initiated treatment with albendazole at a dose of 15 mg/kg/day together with intravenous methylprednisolone at a dose of 1 mg/kg/day for 10 days while continuing sodium valproate 500 mg twice daily. His symptoms improved, with headache resolution, fatigue reduction, and CRP decrease from 126 mg/L to 20 mg/L. Follow-up MRI four months later showed the disappearance of contrast enhancement, consistent with a favorable response. During two years of follow-up, with assessments every three months, he remained seizure-free and returned to work without evidence of clinical recurrence.

Case 2

A 71-year-old woman from a rural area known for boar hunting traditions, with hypothyroidism treated with levothyroxine and no previous history of seizures, was admitted for three brief febrile focal myoclonic seizures involving the left upper limb. Three days earlier, she had developed diffuse myalgia, abdominal pain, and vomiting.

**Figure 2:**
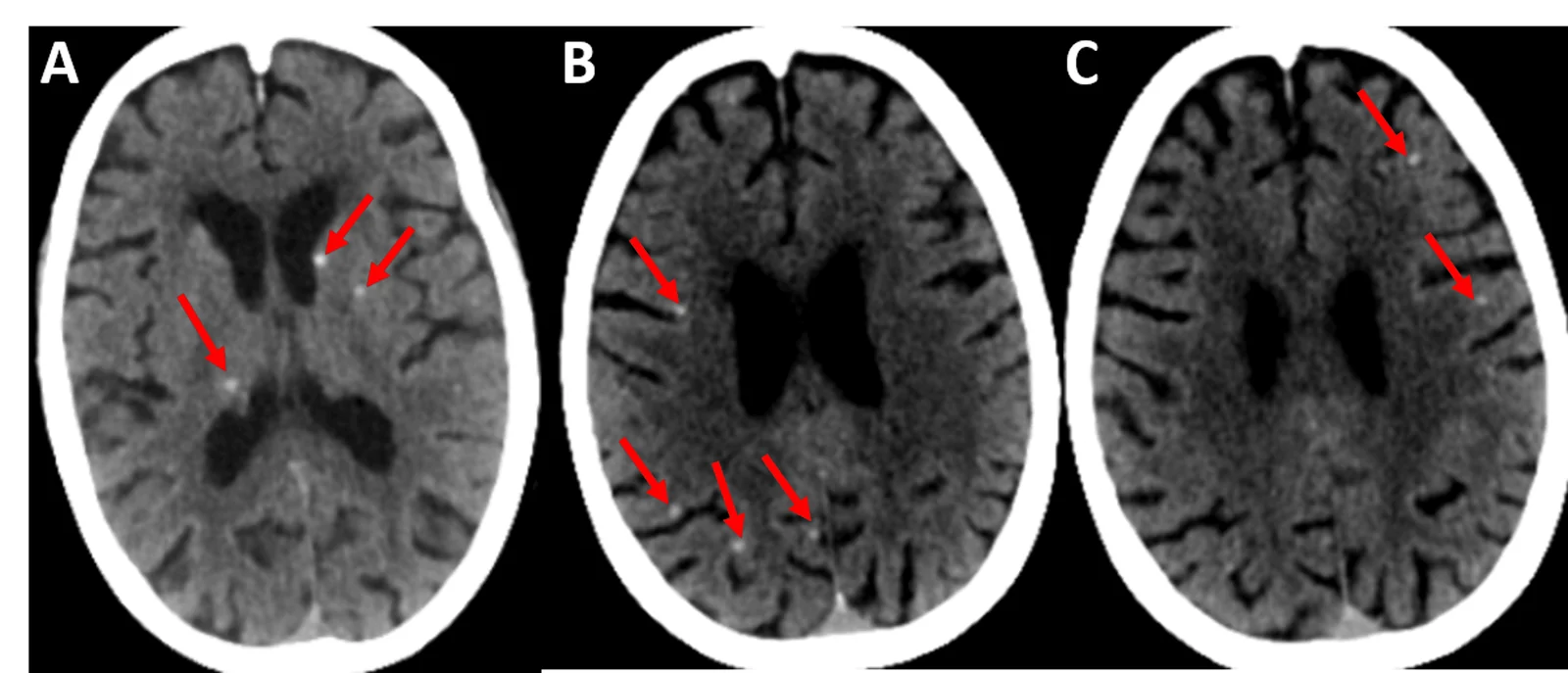
Axial non-contrast brain computed tomography scan showing multiple small hyperdense rounded lesions (A) Left caudate, lentiform nuclei, and right thalamus. (B) Right frontal and parietal cortex. (C) Left frontal cortex.

On admission, she was conscious but nonverbal, asthenic, and febrile, with a body temperature of 39°C. Her blood pressure was 120/70 mmHg, her heart rate was 68 beats/min, and her capillary blood glucose was 0.9 g/L. Neck examination showed no stiffness, and neurological examination and ophthalmological examination, including fundoscopy, were unremarkable. Axial non-contrast brain CT revealed multiple small cortico-subcortical hyperdense lesions raising the possibility of calcified NCC (Figure 2). CSF analysis was normal. Given the clinical and CT findings, the patient was treated with albendazole at a dose of 800 mg/day with intravenous methylprednisolone at a dose of 1 mg/kg/day while further investigations were planned. Unfortunately, she died the following day before brain MRI and serological testing could be performed.

Case 3

A 30-year-old man from a rural area where wild-boar hunting and consumption are common, with no significant medical history, reported that he did not consume wild-boar meat but had close contact with individuals in his community who did. He was admitted in June 2024 after two generalized tonic-clonic seizures, each lasting less than two minutes and separated by a five-hour interval, associated with persistent right-sided hemibody paresthesia. On admission, he was conscious and oriented. His blood pressure was 110/70 mmHg, his heart rate was 89 beats/min, and his capillary blood glucose was 1.04 g/L. He had no neck stiffness. Neurological and ophthalmological examinations, including fundoscopy, were unremarkable. Electroencephalogram (EEG) showed no epileptiform abnormalities.

**Figure 3:**
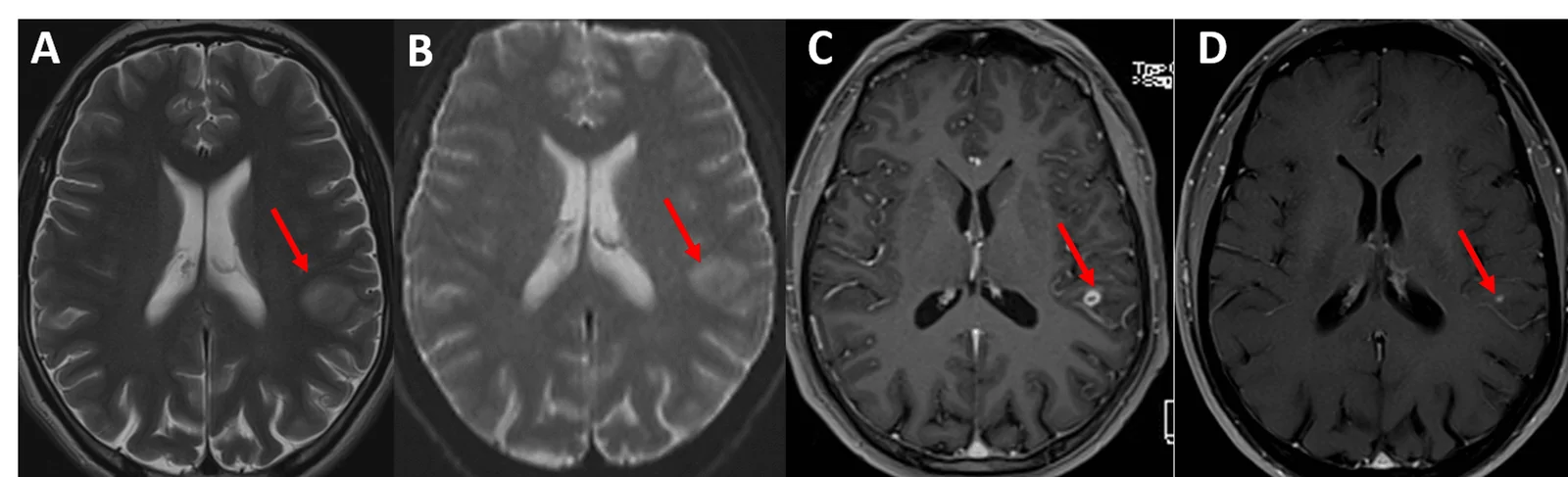
Brain magnetic resonance imaging (A) T2-weighted imaging and (B) diffusion-weighted imaging show a left parietal lesion with surrounding edema. (C) After contrast administration, the left parietal cysticercal lesion demonstrates ring enhancement. (D) Follow-up magnetic resonance imaging performed three months after treatment initiation shows the resolution of the ring enhancement after gadolinium administration.

Brain MRI revealed a parietal ring-enhancing lesion with surrounding edema after gadolinium administration (Figure 3). A granulomatous etiology was initially suspected, and antituberculous therapy was started. However, we discontinued the treatment after CSF PCR testing for *Mycobacterium tuberculosis *returned negative. Investigations for sarcoidosis, toxoplasmosis, and cryptococcosis were also negative.

Follow-up MRI performed one month later showed persistence of the lesion, prompting readmission. Serum testing for TS was positive, supporting the diagnosis of NCC in the colloidal stage. After alternative diagnoses were excluded, a one-week course of albendazole at 15 mg/kg/day and intravenous methylprednisolone at 1 mg/kg/day was initiated because the patient had a single parenchymal lesion in the colloidal phase. Sodium valproate at 500 mg twice daily was continued for seizure control. The clinical course was favorable, with no further seizures and progressive resolution of the right-sided paresthesia. Follow-up MRI three months later showed complete resolution of the ring enhancement and surrounding edema, consistent with a favorable treatment response (Figure 3).

Laboratory and ancillary findings for the three cases are summarized in Table 1.

**Table 1:** Laboratory and ancillary findings of the three cases ELISA: enzyme-linked immunosorbent assay; HIV: human immunodeficiency virus; PCR: polymerase chain reaction; CT: computed tomography

Investigation	Reference range	Case 1	Case 2	Case 3
Blood investigations				
Hemoglobin, g/dL	13-16.5	13.5	14	14.8
Total leukocyte count, ×10³/µL	4.0-10.0	9.0	5.4	8.2
Neutrophils, ×10³/µL	1.5-7.0	6.7	2.3	4.6
Platelets, ×10³/µL	150-400	357	265	226
C-reactive protein, mg/L	<5	126	-	2
Sodium, mEq/L	136-145	143	136	141
Potassium, mEq/L	3.50-5.10	4.30	4.70	4.20
Creatinine, mg/L	7.2-12.5	9.3	13.0	7.3
Aspartate aminotransferase, U/L	5-34	13	-	37
Alanine aminotransferase, U/L	0-55	26	-	44
Serological investigations				
*Taenia solium* ELISA	-	Negative	Not performed	Positive
HIV serology	-	Negative		Negative
Toxoplasma testing	-	Negative		Negative
Cryptococcal testing	-	Negative		Negative
Cerebrospinal fluid investigations				
White cell count, cells/mm³	-	<3	<3	0
Red cell count, cells/mm³	-	<3	<3	0
Protein, g/L	0.15-0.40	0.30	0.40	0.20
Glucose, g/L	0.3-1.0	0.82	0.50	0.58
*Mycobacterium tuberculosis* PCR	-	Negative	Not performed	Negative
Multiplex PCR panel	-	Negative	Not performed	Negative
Sarcoidosis workup				
Serum angiotensin-converting enzyme, U/L	16-85	Not performed	Not performed	73.2
Serum calcium, mg/L	84-102			90
Ophthalmological examination for sarcoidosis	-			No ocular findings suggestive of sarcoidosis
Thoracic and abdominopelvic CT scan	-			No findings suggestive of sarcoidosis
Minor salivary gland biopsy	-			No granulomatous inflammation
Other investigations				
Ophthalmological examination for ocular cysticercosis	-	Negative	Not performed	Negative
Subcutaneous examination/imaging	-	Negative		Not performed

The main clinical events, diagnostic steps, treatments, and outcomes of the three cases are summarized in Table 2.

**Table 2:** Clinical timeline of the three cases CRP: C-reactive protein; CSF: cerebrospinal fluid; CT: computed tomography; EEG: electroencephalogram; ELISA: enzyme-linked immunosorbent assay; MRI: magnetic resonance imaging; PCR: polymerase chain reaction

Time point	Case 1	Case 2	Case 3
Initial event	Six months before admission, the patient had been treated in the intensive care unit for febrile meningoencephalitis with impaired consciousness and generalized seizures	The patient presented with recurrent brief febrile myoclonic seizures of the left upper limb after three days of systemic symptoms, including myalgia, abdominal pain, and vomiting	The patient presented with two generalized tonic-clonic seizures associated with right hemibody paresthesia
Early course/initial assessment	After discharge, he developed persistent diffuse headaches, weight loss, and marked fatigue, without seizure recurrence	Initial examination found fever and asthenia, without meningeal stiffness, focal neurological deficit, or ophthalmological abnormality	Neurological examination was normal, and EEG did not show epileptiform abnormalities
Imaging	MRI revealed multiple cortico-subcortical and thalamic lesions, some containing an eccentric scolex	Emergency CT showed multiple small calcified cortico-subcortical lesions. MRI was planned but could not be performed because of early death	Initial MRI showed a ring-enhancing parietal lesion with surrounding edema; repeat MRI one month later showed lesion persistence
Diagnostic workup	CSF analysis was normal. Tuberculosis PCR, multiplex PCR, and serum *T. solium *ELISA were negative. Ophthalmological and subcutaneous screening showed no extra-neurological involvement	CSF analysis was normal. Serological testing could not be performed before death	Tuberculosis PCR was negative. Workup for sarcoidosis, toxoplasmosis, and cryptococcosis was unrevealing. Serum testing for *T. solium *was positive
Treatment	Albendazole and intravenous corticosteroids were administered for 10 days, in addition to antiseizure therapy	Albendazole, methylprednisolone, and antiseizure treatment were initiated	Albendazole and corticosteroids were administered for one week, with antiseizure therapy
Outcome/follow-up	The patient improved clinically, with the resolution of headaches, reduced fatigue, decreased CRP, and the disappearance of contrast enhancement on follow-up MRI after four months	The patient died the day after admission	The patient improved clinically, with seizure control and regression of paresthesia. Follow-up MRI after three months showed the resolution of enhancement and edema

## Discussion

NCC represents the most common parasitic infection of the central nervous system worldwide [2]. It results from ingestion of TS eggs, either through exogenous contamination, such as contaminated raw produce, or through autoinfection in individuals harboring an intestinal tapeworm due to reverse peristalsis [1,2]. In Morocco, NCC may be overlooked because it is perceived as rare. Nevertheless, it should remain in the differential diagnosis of otherwise unexplained neurological manifestations, particularly seizures, in patients with relevant epidemiological exposure, including residence in or travel to endemic areas or potential fecal-oral exposure to a person with intestinal TS taeniasis [3].

All three patients came from rural communities where wild-boar hunting and meat consumption remain traditional practices. Although domestic pigs are the principal intermediate host of TS [1], wild boars may also harbor its cysticerci [5,6] and may therefore represent a potential source of human taeniasis when infected meat is consumed raw or undercooked. However, direct evidence linking wild-boar meat consumption to confirmed human TS taeniasis remains limited. None of our patients reported consuming wild-boar meat, although they had close contact with community members who did. Thus, indirect fecal-oral transmission from an unidentified TS tapeworm carrier remains the most plausible route of infection.

Clinical presentation varies according to lesion number, location, evolutionary stage, and host inflammatory response [3]. Larvae may localize in the brain parenchyma, ventricles, subarachnoid space, or spinal cord; and lesions evolve through vesicular, colloidal, granular, and calcified stages [3,7]. Seizures represent the most common manifestation of parenchymal NCC and occur in up to 80% of cases, particularly with degenerating cysts [7,8]. Other manifestations include headaches, focal neurological deficits, intracranial hypertension, hydrocephalus, and cognitive or psychiatric symptoms [1]. In our series, all patients presented with seizures, consistent with their predominantly parenchymal involvement. One patient also had chronic headaches, showing that NCC may present with milder or isolated neurological symptoms.

Neuroimaging plays a key role in the diagnosis of NCC [1]. MRI better detects cystic lesions and characterizes their evolutionary phase, whereas CT is particularly useful for identifying calcifications. Visualization of an eccentric scolex, which is pathognomonic of the vesicular phase, constitutes an absolute diagnostic criterion [1,3]. The scolex may appear iso- or hyperintense on conventional T1- and T2-weighted MRI sequences and may demonstrate contrast enhancement, while FLAIR and DWI can further improve its visualization [9]. In our case, imaging demonstrated different evolutionary patterns: the first patient had lesions at various phases, including some with a visible scolex; the second had calcified lesions on CT; and the third had a ring-enhancing lesion suggestive of the colloidal phase. The choice of imaging modality also depended on clinical urgency and local availability. MRI allowed detailed lesion characterization in Cases 1 and 3, whereas Case 2 underwent emergency CT because she was admitted outside MRI working hours. MRI was planned but could not be performed because she died the following day.

Serology may provide supportive evidence, as in our patient with positive ELISA, but its value depends on the assay used and parasite burden [1]. The enzyme-linked immunoelectrotransfer blot (EITB), considered the reference test, has a sensitivity of up to 98% in patients with multiple lesions but is less sensitive in cases with a single lesion, while ELISA has lower sensitivity and specificity [3]. Moreover, serology may be negative in intraparenchymal forms and therefore does not exclude the diagnosis [1,3].

Based on these findings, we established the diagnosis of NCC using the diagnostic criteria proposed by Oscar H. Del Brutto. Under the 2001 criteria [10], the first patient fulfilled the criteria for definitive NCC based on visualization of the scolex. The third patient fulfilled the criteria for probable NCC based on compatible neuroimaging findings, positive serum ELISA for TS, and lesion resolution after antiparasitic therapy. In the second patient, the diagnosis was particularly challenging. Although CT demonstrated multiple parenchymal calcified lesions suggestive of NCC and the patient presented with seizures, brain MRI and serological testing could not be performed because of her early death. Consequently, the diagnosis could not be established with the same degree of certainty as in the other two patients. The revised 2017 criteria [11], which place greater emphasis on neuroimaging findings, did not alter the diagnostic classification of the first and third patients.

Treatment combines symptomatic management with antiparasitic therapy, often associated with corticosteroids to reduce the inflammatory response induced by parasite destruction [1]. In our series, all patients received albendazole, corticosteroids, and antiseizure treatment. Prognosis depends on lesion burden, location, and host inflammatory response. Although parenchymal forms usually have a more favorable outcome than subarachnoid forms, they may still cause severe complications, including status epilepticus or sudden death, as illustrated by the fatal outcome in one of our patients [1,12].

Beyond individual diagnosis and treatment, the occurrence of NCC in regions where it is considered uncommon also raises important public health concerns. In Morocco, where pork consumption is uncommon and published data on NCC remain scarce, the disease is likely underrecognized. However, migration from endemic countries, international travel, persistent sanitation gaps, and rural practices such as wild-boar hunting and meat consumption may provide an epidemiological context in which TS transmission can occur [2,4-6]. In this context, identifying intestinal tapeworm carriers is essential, as they may sustain transmission within households or rural communities when hygiene and sanitation are inadequate [5,6]. These observations highlight the need for better surveillance, carrier detection and treatment, hygiene education, food safety measures, and improved sanitation.

## Conclusions

These observations emphasize the need to consider NCC in non-endemic settings, particularly in patients from rural areas who present with seizures or persistent nonspecific headaches. When CT is normal or inconclusive, brain MRI may reveal diagnostic features such as an eccentric scolex. Serological testing may further support the diagnosis, allowing earlier treatment and helping prevent severe complications.
